# Thermodynamic Costs of Information Processing in Sensory Adaptation

**DOI:** 10.1371/journal.pcbi.1003974

**Published:** 2014-12-11

**Authors:** Pablo Sartori, Léo Granger, Chiu Fan Lee, Jordan M. Horowitz

**Affiliations:** 1 Max Planck Institute for the Physics of Complex Systems, Dresden, Germany; 2 Departamento de Física Atómica, Molecular y Nuclear and GISC, Universidad Complutense de Madrid, Madrid, Spain; 3 Department of Bioengineering, Imperial College London, London, United Kingdom; 4 Department of Physics, University of Massachusetts at Boston, Boston, Massachusetts, United States of America; University of Michigan, United States of America

## Abstract

Biological sensory systems react to changes in their surroundings. They are characterized by fast response and slow adaptation to varying environmental cues. Insofar as sensory adaptive systems map environmental changes to changes of their internal degrees of freedom, they can be regarded as computational devices manipulating information. Landauer established that information is ultimately physical, and its manipulation subject to the entropic and energetic bounds of thermodynamics. Thus the fundamental costs of biological sensory adaptation can be elucidated by tracking how the information the system has about its environment is altered. These bounds are particularly relevant for small organisms, which unlike everyday computers, operate at very low energies. In this paper, we establish a general framework for the thermodynamics of information processing in sensing. With it, we quantify how during sensory adaptation information about the past is erased, while information about the present is gathered. This process produces entropy larger than the amount of old information erased and has an energetic cost bounded by the amount of new information written to memory. We apply these principles to the *E. coli*'s chemotaxis pathway during binary ligand concentration changes. In this regime, we quantify the amount of information stored by each methyl group and show that receptors consume energy in the range of the information-theoretic minimum. Our work provides a basis for further inquiries into more complex phenomena, such as gradient sensing and frequency response.

## Author Summary

The ability to process information is a ubiquitous feature of living organisms. Indeed, in order to survive, every living being, from the smallest bacterium to the biggest mammal, has to gather and process information about its surrounding environment. In the same way as our everyday computers need power to function, biological sensors need energy in order to gather and process this sensory information. How much energy do living organisms have to spend in order to get information about their environment? In this paper, we show that the minimum energy required for a biological sensor to detect a change in some environmental signal is proportional to the amount of information processed during that event. In order to know how far a real biological sensor operates from this minimum, we apply our predictions to chemo-sensing in the bacterium *Escherichia Coli* and find that the theoretical minimum corresponds to a sizable portion of the energy spent by the bacterium.

## Introduction

In order to perform a variety of tasks, living organisms continually respond and adapt to their changing surroundings through diverse electrical, chemical and mechanical signaling pathways, called sensory systems [1]. In mammals, prominent examples are the neurons involved in the visual, olfactory, and somatic systems [2]–[5]. But also unicellular organisms lacking a neuronal system sense their environment: Yeast can sense osmotic pressure [6], and *E. coli* can monitor chemical gradients [7], temperatures [8] and pH [9]. Despite the diversity in biochemical details, sensory adaptation systems (SAS) exhibit a common behavior: long-term storage of the state of the environment and rapid response to its changes [10]. Intuitively, one expects that for these SAS to function, an energy source – such as ATP or SAM – is required; but is there a fundamental minimum energy needed? To tackle this question, we first relate a generic SAS to a binary information processing device, which is tasked to perform fast information acquisition on the environment (response) and to record subsequently the information into its longer term memory (adaptation). Since the foundational works of Maxwell, Szilard and Landauer, the intimate relationship between thermodynamic costs and information processing tasks has been intensely studied [11]–[17]. As a result, the natural mapping between a generic SAS and an information processing device allows us to quantify the minimal energetic costs of sensory adaptation.

The idea of viewing biological processes as information processing tasks is not new [7], [12], [18]. However, rationalizing sensory adaptation is complicated by recent studies that have revealed that motifs in the underlying biochemical networks play a fundamental role in the thermodynamic costs. For instance, the steady state of feedback adaptive systems must be dissipative, with more dissipation leading to better adaptation [19], an observation echoed in the analysis of a minimal model of adaptive particle transport [20]. Other studies have suggested that some feedforward adaptive systems may require dissipation to sustain their steady state [21], while some may not [22], [23]. Furthermore, past studies [18], [24] have approached the notion of information by considering noisy inputs due to stochastic binding, a realm in which adaptation may not be relevant due to the separation of time-scales [25]. Here, we develop a different approach that avoids these caveats by considering a thermodynamically consistent notion of information that naturally incorporates the costs of sensing in sensory adaptation. Specifically, we derive a collection of universal bounds that relate the thermodynamic costs of sensing to the information processed. These bounds reveal for the first time that for a generic SAS, measuring an environmental change is energetically costly [(6) below], while to erase the memory of the past is energetically free, but necessarily irreversible [(5) below]. By formalizing and linking the information processing and thermodynamics of sensory systems, our work shows that there is an intrinsic cost of sensing due to the necessity to process information.

To illustrate our generic approach, we study first a minimal four-state feedforward model and then a detailed ten-state feedback model of *E. coli* chemotaxis. Owing to the symmetry of its motif's topology the four-state feedforward model does not require energy to sustain its adapted state. Instead, all the dissipation arises from information processing: acquiring new information consumes energy, while erasing old information produces entropy. By contrast, the *E. coli* model sustains its nonequilibrium steady state (NESS) by constantly dissipating energy, a requirement for adaptation with a feedback topology [19]. In this nonequilibrium setting, we generalize our thermodynamic bounds in order to pinpoint the additional energy for sensing over that required to maintain the steady state. We find with this formalism that in *E. coli* chemotaxis the theoretical minimum demanded by our bounds accounts for a sizable portion of the energy spent by the bacterium on its SAS.

## Results

### Universal traits of sensory adaptation

To respond and adapt to changes in an environmental signal 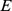, a SAS requires a fast variable, the activity 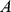; and a slow variable, the memory 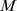. For example, in *E. coli* the activity is the conformational state of the receptor, the memory the number of methyl groups attached to it, and the signal is the ligand concentration [7]. Without loss of generality, we consider in the following all three variables normalized such that they only lie between 0 and 1, and that the signal can only alternate between two values: a low value 0 and a high value 1.

As a result of thermal fluctuations, the time-dependent activity 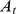 and memory 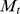 are stochastic variables. Yet, the defining characteristics of sensory adaptation are captured by their ensemble averages 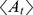 and 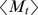, both at the steady state and in response to changes in the signal.

At a constant environmental signal 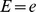, the system relaxes to an adapted 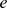-dependent steady state, which may be far from equilibrium [19]. In this state, the memory is correlated with the signal, with an average value close to the signal, 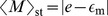 where 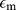 is a small error. The average activity however is *adapted*, taking a value roughly independent of the signal, 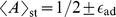, with adaption error 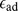.

**Figure 1:**
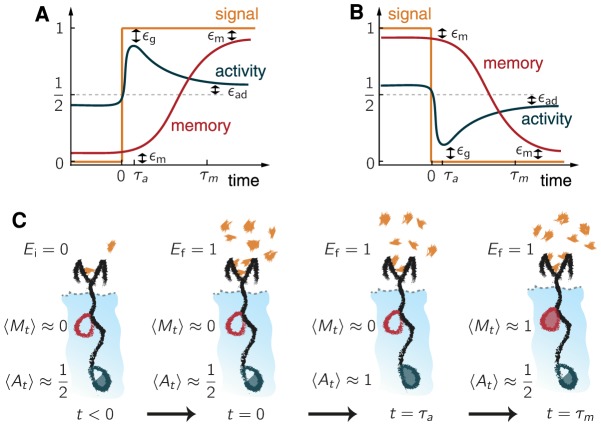
Generic traits of sensory adaptive systems. (A/B) Typical time evolution of the average activity 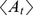 (dark blue) and average memory 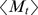 (red) of a SAS in response to an abrupt increase or decrease in the signal 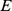 (orange). (C) Schematic states of a chemical receptor (black) embedded in a cell (light blue) during the four key phases of adaptation. At 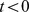 the system is adapted; at 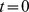 there is a sudden increase in the signal ligand concentration (orange flecks); at 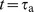 the receptor responds increasing its activity (full blue circle); and at time 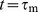 it is adapted (the memory is full, red; while the activity is half full blue).

Besides the ability to adapt, SAS are also defined by their multiscale response to abrupt signal changes, which is illustrated in Fig. 1. For example, given a sharp increase in the signal from 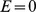 to 1 the average activity quickly grows from its adapted value to a peak 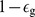 characterized by the gain error 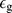. This occurs in a time 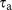, before the memory responds. After a longer time 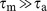, the memory starts to track the signal, and the activity gradually recovers to its adapted value (see Fig. 1A). For a sharp decrease in the signal, the behavior is analogous (see Fig. 1B).

We identify a SAS as any device that exhibits the described adapted states for low and high signals (0 or 1) and that reproduces the desired behavior to abrupt increases and decreases in the signal (see Fig. 1C for a cartoon biochemical example). While SAS typically exhibit additional features (such as wide range sensitivity [26], [27]), they all exhibit the universal features illustrated in Fig. 1.

### Minimal SAS: Equilibrium feedforward model

**Figure 2:**
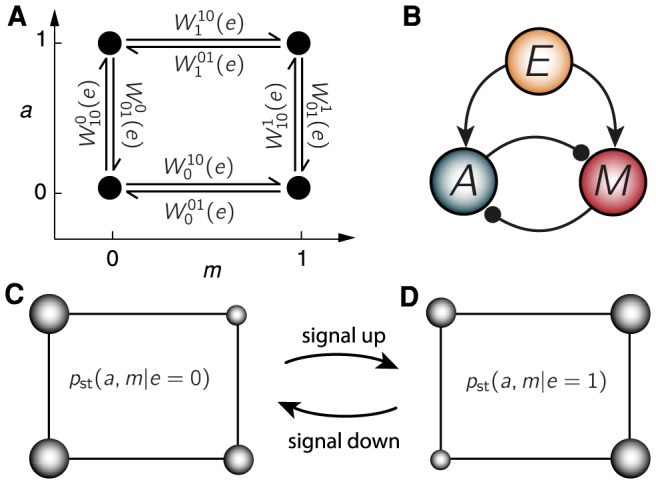
Equilibrium adaptation in a symmetric feedforward SAS. (A) Reaction network of the four states in activity, 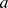, memory, 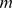, space, with kinetic rates 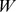 indicated for each transitions. (B) Topology of the model: feedforward with mutual inhibition. For a fixed signal 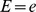, a sudden increase in the memory makes the average activity drop, and vice versa for activity changes. This symmetry of the topology, which is at the core of detailed balance, allows an equilibrium construction. (C/D) Representation of steady state probabilities 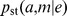 for low/high 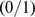 signals using the 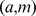 space in (A). Wider state diameter represents higher probability, thus lower energy.

To facilitate the development of our formalism, we first present a minimal stochastic model of a SAS, where the activity 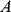 and memory 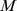 are binary variables (0 or 1). This model is minimal, since it has the least number of degrees of freedom (or states) possible and still exhibits the required response and adaptive behavior. Treating the environmental signal 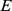 as an external field that drives the SAS, the system can be viewed as evolving by jumping stochastically between its four states depicted in Fig. 2A. The rates for activity 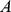 transitions from 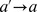 given 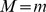 at fixed 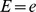 are denoted 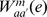, and those for memory 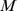 transitions from 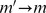 given 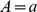 are 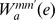.

As an equilibrium model, it is completely characterized by a free energy function, which we have constructed in the Methods by requiring the equilibrium steady state to have the required signal correlations of a SAS,
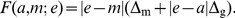
(1)



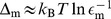
 is the energy penalty for the memory to mistrack the signal, ensuring adaptation (with 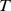 the temperature and 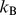 Boltzmann's constant). In fact, one can show that 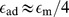. 
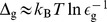
 is the penalty for the activity to mistrack the signal when 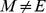; it thus becomes relevant after a signal change, but before the memory adapts to the new signal, ensuring response. In Figs. 2C and D the energy landscape 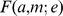 is represented for low and high signals (smaller radius corresponds to less probability and larger energy). Note that for fixed 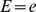, the adaptation error is zero when the energy penalty to misstrack the signal becomes large 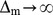, the system's configuration is then 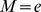 and 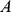 takes on the values 0 and 1 with equal probability. Finally, the dynamics are set by fixing the kinetic rates using detailed balance, *e.g.*, 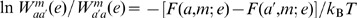, and then choosing well-separated bare rates to set the timescale of jumps: 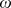 for activity transitions and 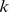 for memory transitions, with 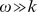, thereby enforcing the well-separated time-scales of adaptation.

When there is a change in the signal, this model exhibits response and adaptation as characterized in Figs. 1A and B (verified in S1 and S2 Figures), and relaxes towards a *dissipationless* equilibrium steady state in which detailed balance is respected. This is in contrast to previous studies on adaptive systems, which demonstrated that maintaining the steady state for a generic feedback system breaks detailed balance [19], [20]. Our model, however, differs by its network topology. As depicted in Fig. 2B, it is a mutually repressive feedforward (all rates depend explicitly on 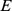, and the actions of 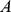 and 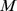 on each other are symmetric). Similar topologies also underly recent suggestions for biochemical networks that allow for adaptation with dissipationless steady states [22], [23].

### Information processing in sensory adaptation

Any sensory system that responds and adapts can naturally be viewed as an information processing device. In the steady state, information about the signal is stored in the memory, since knowledge of 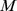 allows one to accurately infer the value of 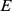. The activity 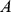, on the other hand, possesses very little information about the signal, since it is adapted and almost independent of the signal. When confronted by an abrupt signal change, the activity rapidly responds by gathering information about the new signal value. As the activity decays back to its adapted value, information is stored in the memory. However, to make room for this new information, the memory must decorrelate itself with the initial signal, thereby erasing the old information. Thus sensory adaptation involves measurement as well as erasure of information.

To make this intuitive picture of information processing precise, let us focus on a concrete experimental situation where the signal is manipulated by an outside observer. This is the setup common in experiments on *E. coli* chemotaxis where the signal (the ligand concentration) is varied in a prescribed, deterministic way [28]. To be specific, the initial random signal 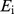 is fixed to an arbitrary value 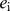, either 0 or 1, with probability 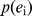, and the system is prepared in the corresponding 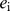-dependent steady state, characterized by the probability density 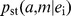. Then, at time 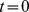, the signal is randomly switched to 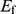 with final value 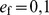 (which may be the same as 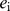) according to the probability 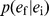. The signal is held there while the system's time-dependent probability density 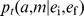, which conditionally depends on both the initial and final signals, irreversibly relaxes to the final steady state 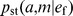. During this relaxation correlations between the system and the final signal value 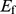 develop while the correlations with the past value 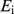 are lost. As we will see, the measure of information that captures this evolution of correlations *and* naturally enters the thermodynamics of sensory adaptation is the mutual information between the system and the signal.

The *mutual information* is an information-theoretic quantification of how much a random variable 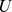 (such as the system) knows about another variable 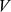 (such as the signal),
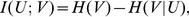
(2)measured in nats [29]. Here, 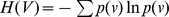 is the Shannon entropy, which is a measure of uncertainty. Thus, the mutual information measures the reduction in uncertainty of one variable given knowledge of the other. Of note, 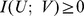 with equality only when 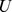 and 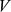 are independent.

There are two key appearances of mutual information in sensory adaptation capturing how information about the present is acquired, while knowledge of the past is lost, which we now describe. At the beginning of our experiment at 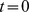, the SAS is correlated with 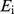, simply because the SAS is in a 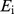-dependent steady state. Thus there is an initial information 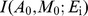 that the SAS has about the initial value of the signal 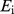. The signal is then switched; yet immediately after, the SAS has no information about the new signal value 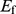, so 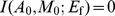. Then for 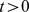 the SAS evolves, becoming correlated with 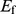, thereby gathering (or measuring) information 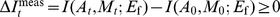, which grows with time. Concurrently it decorrelates from 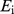, thus erasing information 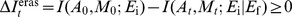 about the old signal, which also grows with time. This conditioning 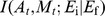 only takes into account direct correlations between 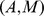 and 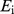, excluding indirect ones through 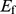.

**Figure 3:**
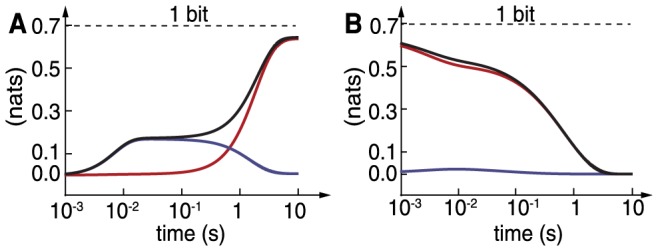
Information measurement and erasure in sensory adaptation. (A) Information acquired about the new signal as a function of time. The information stored in the activity 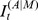 (dark blue) grows as the system responds, and then goes down as it adapts, when the information in the memory 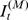 (red) grows. The total information measured 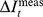 (black) shows the effect of both. (B) Information lost about the old signal 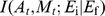 (black), and its decomposition in memory (red) and activity (blue) information. Model parameters are 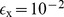 for x = a, m, g; 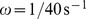 and 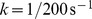.

To illustrate this, we calculate the flow of information in the non-disspative feedforward model for 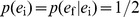, which is a 1-bit operation (because 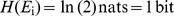). Fig. 3A displays the evolution of the measured information (in black), which we decomposed as
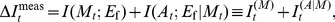
(3)where 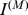 (red) is the information stored in the memory and 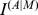 (blue) in the activity. We see the growth of 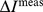 proceeds first by a rapid (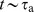) increase as information is stored in the activity (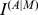 grows) while the system responds, followed by a slower growth as adaptation sets in (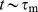), and the memory begins to track the signal. At the end, the system is adapted, and there is almost no information in the activity, 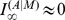. With the small errors we have, the information acquired reaches nearly the maximum value of 1 bit, which is stored in the memory 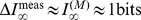. Fig. 3B shows the erasure of information, visible by the decrease of 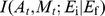 from an initial value of nearly one bit to zero when the system has decorrelated from the initial signal 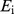.

### Thermodynamic costs to sensory adaptation

We have seen that through an irreversible relaxation, an SAS first acquires and then erases information in the registry of the activity, followed by the memory. The irreversibility of these information operations is quantified by the entropy production, which we now analyze in order to pinpoint the thermodynamic costs of sensing. Specifically, we demonstrate in Methods that for a system performing sensory adaptation in response to an abrupt change in the environment, the total entropy production can be partitioned in two positive parts: one caused by measurement (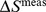) and the other by erasure (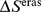). The second law thus becomes
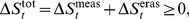
(4)with the reference set to an initial state at 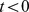. The erasure piece
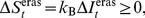
(5)is purely entropic in the sense that it contains no energetic terms. It solely results from the loss of information (or correlation) about the initial signal. By contrast, the energetics are contained in the measurement portion,
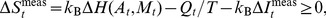
(6)where 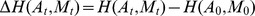 is the change in Shannon entropy of the system and 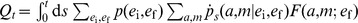 is the average heat flow into the system from the thermal reservoir.

A useful alternative formulation can be obtained once we identify the internal energy 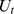. For example, in the equilibrium feedforward model, a sensible choice is the average energy 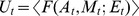 (1). (Recall, that there is no unique division into internal energy and work, though any choice once made is thermodynamically consistent [30], [31].) By substituting in the first law of thermodynamics 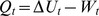, with 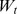 the work, we arrive at
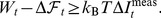
(7)


This equation shows how the measured information 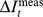 bounds the minimum energy required for sensing, which must be supplied as either work 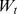 or free energy 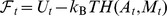. Thus, *to measure is energetically costly; whereas, erasure is energetically free, but necessarily irreversible.* In particular, for sensing to occur, the old information must be erased (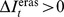), implying that the process is inherently irreversible,
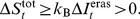
(8)


Together (5) and (7) quantify the thermodynamic cost of sensing an abrupt change in the environment by an arbitrary sensory system.

We have demonstrated from fundamental principles that sensing generically requires energy. However, (7) does not dictate the source of that energy: It can be supplied by the environment itself or by the SAS. The distinction originates because the definition of internal energy is not unique, a point to which we come back in our analysis of *E. coli* chemotaxis.

**Figure 4:**
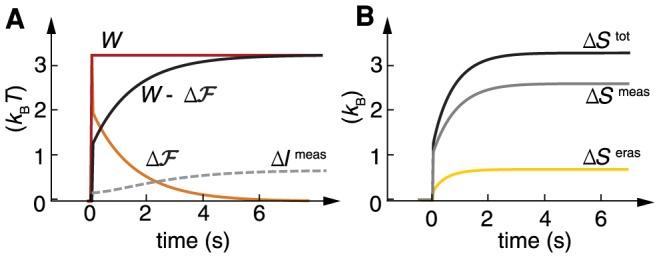
Thermodynamics of adaptation in an equilibrium SAS. (A) Energetic cost as a function of time given by the work 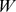 provided by the environment (red), free energy change of the system 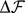 (orange), and dissipated work 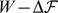 (black), compared to the measured information 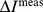 (grey dashed), which gives the lower bound at every time. (B) Total entropic cost 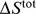 (black) and decomposition in measurement 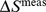 (gray) and erasure 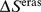 (yellow). Parameters as in Fig. 3.

Using again our equilibrium feedforward model as an example, we apply our formalism to investigate the costs of sensory adaptation. Since this model sustains its steady state at no energy cost, the ultimate limit lies in the sensing process itself. We see this immediately in Fig. 4 where we verify the inequalities in (4) and (7). Since 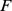 in (1) is explicitly a function of the environmental signal 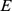, the sudden change in 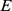 at 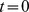 does work on the system, which is captured in Fig. 4A by the initial jump in 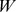. This work is instantaneously converted into free energy 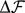 and is then consumed as the system responds and adapts in order to measure. Thus, in this example the work to sense is supplied by the signal (the environment) itself and not the SAS, which is consistent with other equilibrium models of SAS [23]. Furthermore, Fig. 4B confirms that the erasure of information leads to an irreversible process with net entropy production. The bounds of (4) and (7) are not tightly met in our model, since we are sensing a sudden change in the signal that necessitates a dissipative response. Nonetheless, the total entropy production and energetic cost are on the order of the information erased and acquired. This indicates that these information theoretic bounds can be a limiting factor for the operation of adaptive systems. We now show that this is the case for *E. coli* chemotaxis, a fundamentally different system as it operates far from equilibrium.

### Extension to NESS and application to *E. coli* chemotaxis

We have quantified the thermodynamic costs in any sensory adaptation system; however, for systems that break detailed balance and maintain their steady state far from equilibrium, (5) – (8) are uninformative, because of the constant entropy production. A case in point is *E. coli*'s SAS, which enables it to perform chemotaxis by constantly consuming energy and producing entropy through the continuous hydrolysis of SAM.

Nevertheless, there is a refinement of the second law for genuine NESS in terms of the nonadiabatic 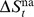 and adiabatic 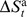 entropy productions, 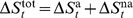 [32]. Crudely speaking, 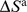 is the entropy required to sustain a nonequilibrium steady state and is never null for a genuine NESS; whereas 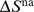 is the entropy produced by the transient time evolution. When the system satisfies detailed balance 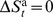 always, be it at its equilibrium steady state or not; when its surroundings change, the entropy production is entirely captured by 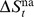. We can refine our predictions for a NESS by recognizing that 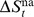 captures the irreversibility due to a transient relaxation, just as 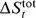 does for systems satisfying detailed balance. Analogously to Eqs. (6) and (8), we derive (see Methods):
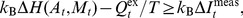
(9)

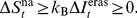
(10)


Here, 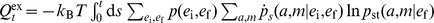 is the excess heat flow into the system, roughly the extra heat flow during a driven, nonautonomous process over that required to maintain the steady state [33]. As a result, it remains finite during an irreversible relaxation to a NESS, even though the NESS may break detailed balance.

**Figure 5:**
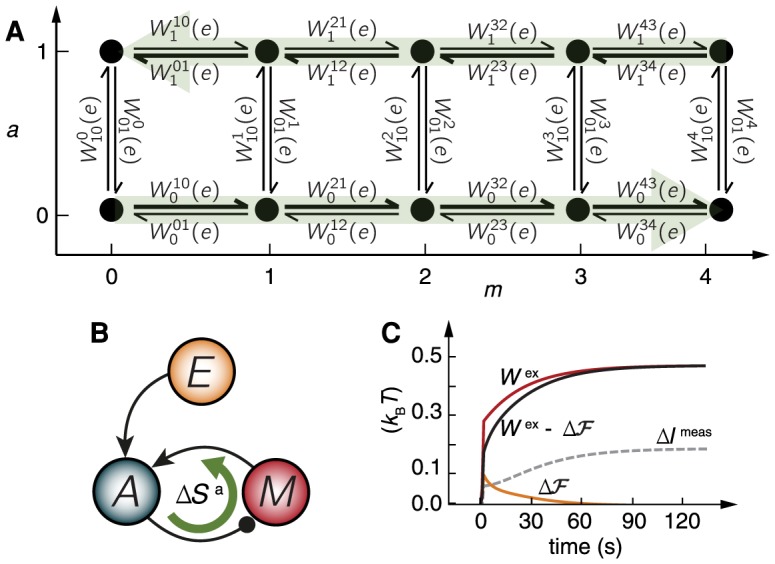
Energetic costs of adaptation in an *E. coli* chemotaxis SAS. (A) Network representation of the nonequilibrium receptor model with five methylation and two activity states. Green arrows represent the addition/removal of methyl groups driven by the chemical fuel SAM. (B) Corresponding negative feedback topology, displaying the dissipative energy cycle (green arrow) sustained by adiabatic entropy production, due to the consumption of chemical fuel. (C) Energetics of nonequilibrium measurement in the chemotaxis pathway for a ligand concentration change of 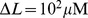 (other parameters in Materials and Methods). The instantaneous change in ligand concentration performs chemical work on the cell, which increases its free energy 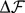 as the cell responds. To adapt, the bacterium has to provide excess work 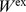 from its own chemical reservoir, the fuel SAM.

*E. coli* is a bacterium that can detect changes in the concentration of nearby ligands in order to perform chemotaxis: the act of swimming up a ligand attractor gradient. It is arguably the best studied example of a SAS. At a constant ligand concentration 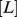, chemoreceptors in *E. coli* – such as the one in Fig. 1C – have a fixed average activity, which through a phosphorylation cascade translates into a fixed switching rate of the bacterial flagellar motor. When 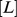 changes, the activity of the receptor 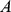 (which is a binary variable labeling two different receptor conformations) increases on a time-scale 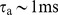. On a longer time-scale 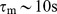, the methylesterase CheR and methyltransferase CheB alter the methylation level of the receptor in order to recover the adapted activity value. In this way, the methylation level 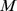 (which ranges from none to four methyl groups for a single receptor) is a representation of the environment, acting as the long-term memory (see diagram in Fig. 5A). One important difference with the previous equilibrium model is that the chemotaxis pathway operates via a feedback. The memory is not regulated by the receptor's signal, but rather by the receptor's activity (see motif in Fig. 5B). The implication is that energy must constantly be dissipated to sustain the steady state [19], thus (9) and (10) are the appropriate tools for a thermodynamic analysis.

There is a consensus kinetic model of *E. coli* chemoreceptors [7], [27], [34]–[36] whose biochemical network is in Fig. 5A. The free energy landscape of the receptor coupled to its environment is
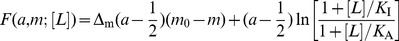
(11)

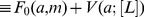
(12)with 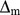 the receptor's characteristic energy, 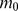 the reference methylation level, and 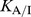 the active/inactive dissociation constants (values in Methods). In (11) the first term 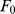 corresponds to the energy of the receptor, and the second 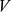 comes from the interaction with the environment (*de facto* a ligand reservoir). The dynamics of this receptor consist of thermal transitions between the states with different activity, while transitions between the different methylation levels are powered by a chemical potential gradient 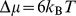 due to hydrolisis of the methyl donor SAM (see Methods). Continuous hydrolysis of SAM at the steady state sustains the feedback at the expense of energy, allowing accurate adaptation in the ligand concentration range 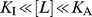, see Fig. 5B.

To begin our study, we develop an equation analogous to (7), which requires identifying the internal energy of our system. As stated above, we consider the binding and unbinding of ligands as external stimuli, and thus define the internal energy as 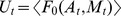. Using the excess heat 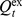, we consistently define the excess work through 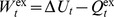, analogous to the first law. Upon substitution into (9) gives
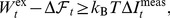
(13)showing just as in (7) that measuring requires excess work and free energy. Because here the internal energy 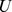 is *not* a function of the ligand concentration, 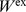 is not due to signal variation: It represents the energy expended by the cell to respond and adapt to the external chemical force.

In Fig. 5C, we compare 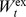 and 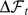 to 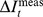 during a ligand change of 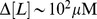. The sudden change in 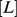 produces a smooth, fast (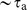) increase in the free energy as the activity transiently equilibrates with the new environment. The excess work driving this response comes mainly from the interaction with environment. As adaptation sets in (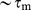), the receptor utilizes that stored free energy, but in addition burns energy by the consumption of SAM. Thus, in order to adapt the cell consumes the free energy stored from the environment, as well as additional excess work coming now mostly from the hydrolysis of SAM molecules. The inequality in (7) with the measured information is satisfied at all times.

The energetic cost of responding and adapting to the ligand change is roughly 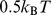, of which much has already been used by 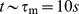. In comparison, the cost to sustain the chemotaxis pathway during this time is roughly 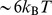 (see Methods). This means that the cost to sensing a step change is about 10% of the cost to sustain the sensing apparatus at steady-state. During this process the cell measures (and erases) roughly 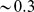 bits, less than the maximum of 1 bit despite its very high adaptation accuracy. This limitation comes from the finite number of discrete methylation levels, so that the probability distributions in *m*-space for large and low ligand concentrations have large overlaps (S3 Figure). In other words, it is difficult to discriminate these distributions, even though the averages are very distinct, which results in lower correlation between the methylation level and signal. The minimal energetic cost associated to measuring these 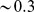 bits (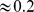 nats) is 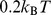. *E. coli* dissipates roughly 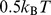 during this process, thus the energetic cost of sensory adaptation is slightly larger than twice its thermodynamic lower bound (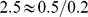).

**Figure 6:**
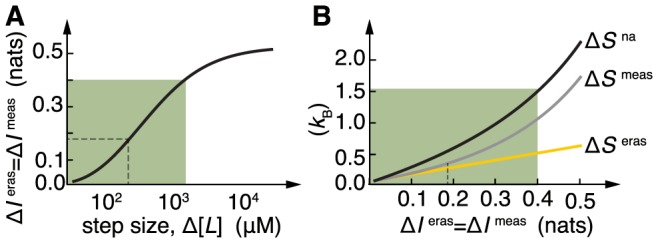
Information-dissipation trade-off in *E. coli* chemotaxis. (A) Relationship between information erased/acquired and size of the signal increase. Shaded in green is the region of accurate adaptation (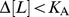). (B) Entropy production as a function of information erased/acquired as step size is varied. The more information is processed by the cell the higher the entropic cost. Notice the linear scaling between dissipation and information for small information (small ligand changes). Dashed lines refer to values in Fig. 5C. Parameters as in Methods.

We further explored the cost of sensing in *E. coli* by examining the net entropy production for ligand changes of different intensity. In Fig. 6A, we plot the amount of information erased/measured for different step changes of the signal up to 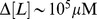 taking as lower base 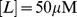. The green shading highlights the region where adaptation is accurate (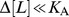). The information erased is always below 1 bit and saturates for high ligand concentrations, for which the system is not sensitive. The total entropic cost (that is, 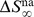) and its relation with the information erased appears in Fig. 6B. The dependence is monotonic, and thus reveals a trade-off between information processing and dissipation in sensory adaptation. Notably, for small acquisition of information (small ligand steps) it grows linearly with the information, an effect observed in ideal measurement systems [17].

## Discussion

We have derived generic information-theoretic bounds to sensory adaptation. We have focused on response-adaptive sensory systems subject to an abrupt environmental switch. This was merely a first step, but the procedure we have outlined here only relies on the validity of the second law of thermodynamics, and therefore can be extend to any small system affected by a random external perturbation to which we can apply stochastic thermodynamics, which is reviewed in [37].

Our predictions are distinct from (although reminiscent of) Landauer's principle [11], [12], which bounds the minimum energy required to reset an isolated memory. By contrast, the information erased in our system is its correlations with the signal. There is another important distinction from the setup of Landauer, and more broadly the traditional setup in the thermodynamics of computation [11] as well as the more recent advancements on the thermodynamics of information processing in the context of measurement and feedback [15], [38]–[45]. There the memory is reset by changing or manipulating it by varying its energy landscape. In our situation, the erasure comes about because the signal is switched. The loss of correlations is stimulated by a change in the measured system – that is the environmental signal; erasure does not occur because the memory itself is altered. Also relevant is [46], which addresses the minimum dissipated work for a system to make predictions about the future fluctuations of the environmental signal, in contrast to the measured information about the current signal, which we have considered.

Our results predict that energy is required to sense changes in the environment, but do not dictate that source of energy. Our equilibrium feedforward model is able to sense and adapt by consuming energy provided by the environment. *E. coli*'s feedback, however, uses mostly external energy to respond, but must consume energy of its own to adapt. The generic bounds here established apply to these two distinct basic topologies, irrespective of their fundamentally different energetics. For *E. coli*, to quantify to what extent 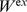 is affected by SAM consumption and ligand binding, a more detailed chemical model is required in conduction with a partitioning of the excess work into distinct terms. An interesting open question in this regard, is why nature would choose the dissipative steady state of *E. coli*, when theoretically the cost of sensing could be paid by the environment.

For a ligand change of 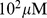, in the region of high adaptation, the information measured/erased is 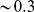 bits. We observed that the corresponding average change in the methylation level for a chemoreceptor is 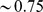, suggesting that a methylation level can store 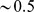 bits for such 1-bit step response operations. Despite the small adaptation error, information storage is limited by fluctuations arising from the finite number of discrete methylation levels. Receptors' cooperativity, which is known to reduce fluctuations of the collective methylation level, may prevent this allowing them to store more information. On the energetic side, we have shown that the cost of sensing these ligand changes per receptor is around 10% of the cost of sustaining the corresponding adaptive machinery. We also showed that the energetic cost of binary operations is roughly twice beyond its minimum for large ligand changes, in stark contrast with everyday computers for which the difference is orders of magnitude. Taken together these numbers suggest that 5% of the energy a cell uses in sensing is determined by information-thermodynamic bounds, and is thus unavoidable.

Future work should include addressing sensory adaptation in more complex scenarios. One which has recently aroused attention is fluctuating environments, which so far has been addressed using trajectory information [44], [45], [47]. However, under physiological conditions this is unlikely to play a significant role given the large separation of time-scales between binding, response, and adaptation [25]. Another scenario is a many bits step operation, in which instead of high and low signals a large discrete set of ligand concentrations is considered. Frequency response and gradient sensing are also appealing [27], since in them the system is in a dynamic steady state in which the memory is continuously erased and rewritten. Analysis of such scenarios is far from obvious, but the tools developed in this work constitute the first step in developing their theoretical framework.

## Methods

### Kinetics of equilibrium feedforward model

We determine a collection of rates that exhibit response and adaptation as in Fig. 1 by first decomposing the steady state distribution as 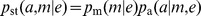. As a requirement to show adaptation, the memory must correlate with the signal, which we impose by fixing 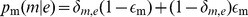. Next, in the steady state the activity is 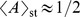, or since 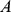 is binary the probability 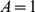 is about 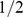. Recognizing that 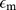 is small, the average 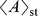 is dominated by adapted configurations with 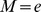. Thus, adaption will occur by demanding that 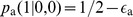 and 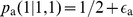, with a model parameter 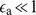. Finally, to fix the activity distribution for non-adapted configurations, 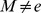, we exploit the time-scale separation 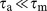. In this limit, after an abrupt change in the signal, the activity rapidly relaxes. To guarantee the proper response, we set 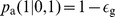 and 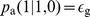. Using the symmetry condition 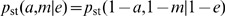 we complete knowledge of 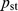. The energy levels 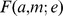 are obtained using the equilibrium condition 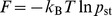, where we choose as reference 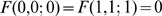. Equation (1) is an approximation of this energy to lowest order in the small errors. Finally, the kinetic rates are obtained using either the approximate or exact energy function, imposing detailed balance, and keeping two bare rates, 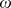 and 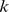, for activity and memory transitions: 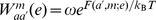 for activity transitions and 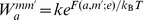 for memory transitions.

### Information bounds on the thermodynamics of sensory adaptation

The bounds in (5) and (6) follow from a rearrangement of the second law of thermodynamics [48]. Consider a system with states 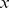 [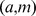 for SAS] with signal-dependent (free) energy function 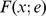 in contact with a thermal reservoir at temperature 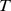. The system is subjected to a random abrupt change in the signal. Specifically, the initial signal is a random variable 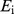 with values 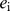 (which are 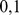 in the main text), which we randomly change at 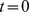 to a new random signal 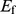 with values 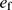. For times 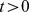, we model the evolution of the system's stochastic time-dependent state 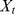 as a continuous-time Markov chain.

We begin our analysis by imagining for the moment that the signal trajectory is fixed to a particular sequence 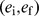. Then our thermodynamic process begins prior to 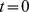 by initializing the system in its 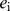-dependent steady state 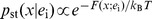. At 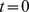, the signal changes to 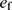 and remains fixed while the system's probability density 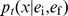, which conditionally depends on the *entire* signal trajectory, evolves according to the master equation [49]

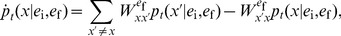
(14)where 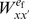 is the signal-dependent transition rate for an 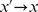 transition. The transition rates are assumed to satisfy a local detailed balance condition, 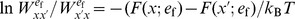, which allows us to identify the energy exchanged as heat with the thermal reservoir in each jump. Eventually, the system relaxes to the steady state 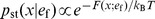 corresponding to the final signal value 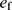.

Since the signal trajectory is fixed, this process is equivalent to a deterministic drive by an external field, and therefore the total entropy production rate will satisfy the second law [48]

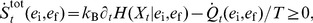
(15)where 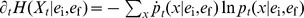 is the rate of change of the Shannon entropy of the system conditioned on the entire signal trajectory; and
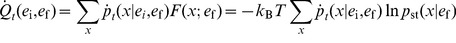
(16)is the heat current into the system from the thermal reservoir given the signal trajectory. Since (15) holds for any signal trajectory, it remains true after averaging over all signal trajectories sampled from the probability density 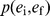:
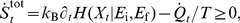
(17)with 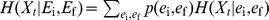, and nonconditioned thermodynamic quantities, such as 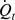, denote signal averages. We next proceed by two judicious substitutions of the definition of the mutual information (2) that tweeze out the contributions from the measured and erased information. First, we replace the Shannon entropy rate as 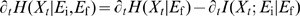, and then immediately repeat 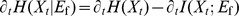. The result is a splitting of the total entropy production rate as 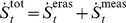, with one part due to erasure
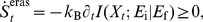
(18)and one due to measurement



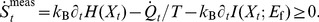
(19)The bounds in (5) and (6) follow by integrating (18) and (19) from time 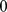 to 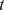.

To prove the positivity of (18) and (19), we use the definition of entropy and heat to recast them in terms of a relative entropy 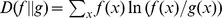 [29] as
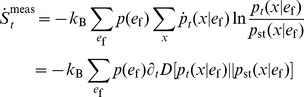
(20)

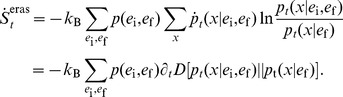
(21)


Positivity then follows, since the relative entropy decreases whenever the probability density evolves according to a master equation, as in (14) [50].

To arrive at (9) and (10) for genuine NESS, we repeat the analysis above applied to the average nonadiabatic entropy production rate (cf. (17))
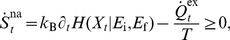
(22)where 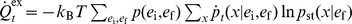 is the excess heat flow into the system [33], taking special note that now 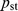 is the nonequilibrium stationary state and cannot be related to the energy, as in the equilibrium case above (16).

### Description of the chemotaxis model

The parameters for 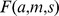 in (11) are taken from [7] for a Tar receptor: 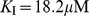, 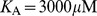, 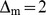, 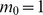. The kinetic rates are obtained using local detailed balance and restricting to two characteristic time-scales. For 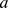-transitions, the rates are 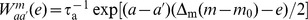, with 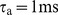 the typical activation time. For 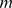-transitions, the rates for active states are 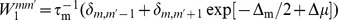, and for inactive states, 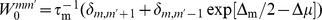. Here, 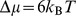 is the chemical potential force for the hydrolyzation of a SAM fuel molecule, which occurs when a methyl group is added or removed by CheR and CheB respectively [19], and at the steady state 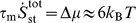.

## Supporting Information

S1 Figure **Adaptation in equilibrium feedforward SAS to a step increase.** Time evolution of average activity (left) and memory (right) during an increase from 0 to 1 of the environmental signal at time *t = 0* for the equilibrium feed-forward model.
(PDF)

S2 Figure **Adaptation in equilibrium feedforward SAS to a step decrease.** Time evolution of average activity (left) and memory (right) during a decrease from 0 to 1 of the environmental signal at time *t = 0* for the equilibrium feed-forward model.
(PDF)

S3 Figure **Probability distributions of the methylation level for low and high signals.** Probability distribution of methylation levels for low (orange) and high (blue) ligand concentration levels in the chemotaxis pathway. To the left, ligand concentrations of *[L] = 94µM* and *[L] = 720µM* were used, which are in the adaptive region *K_I_<<L<<K_A_*. To the right ligand concentrations of *[L] = 720µM* and *[L] = 5760µM*, outside the adaptive region. Notice the large overlap of the distributions. This effect reduces the memory capacity of *E. coli*.
(PDF)
