# Supplementary material for: Thermodynamic Costs of Information Processing in Sensory Adaptation
Source: PLoS Comput Biol. 2014 Dec 11;10(12):e1003974. doi: 10.1371/journal.pcbi.1003974 (PMC4263364; doi:10.1371/journal.pcbi.1003974)

Figure S1. Adaptation in equilibrium feedforward SAS to a step increase. Time evolution of average activity (left) and memory (right) during an increase from 0 to 1 of the environmental signal at time *t=0* for the equilibrium feed-forward model.

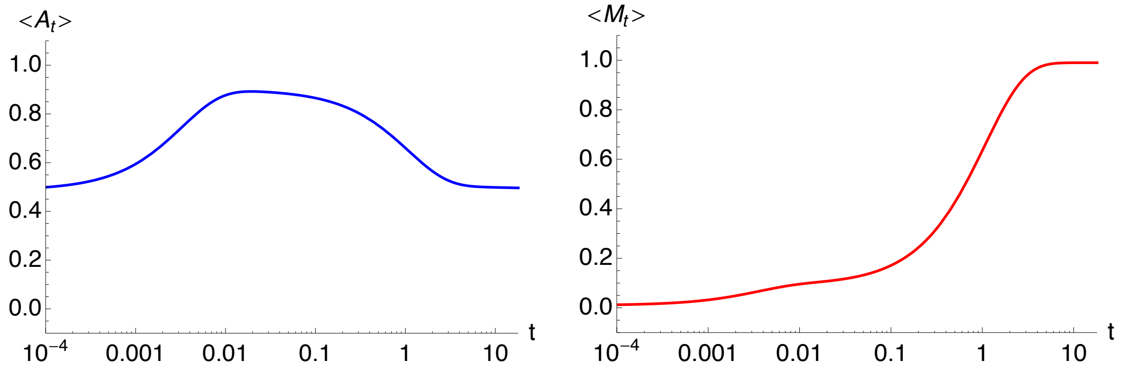

Supplement: S1 Figure — Adaptation in equilibrium feedforward SAS to a step increase. Time evolution of average activity (left) and memory (right) during an increase from 0 to 1 of the environmental signal at time t = 0 for the equilibrium feed-forward model. (PDF) [file pcbi.1003974.s001.pdf]
