# Supplementary material for: Thermodynamic Costs of Information Processing in Sensory Adaptation
Source: PLoS Comput Biol. 2014 Dec 11;10(12):e1003974. doi: 10.1371/journal.pcbi.1003974 (PMC4263364; doi:10.1371/journal.pcbi.1003974)

Figure S3. Probability distributions of the methylation level for low and high signals. Probability distribution of methylation levels for low (orange) and high (blue) ligand concentration levels in the chemotaxis pathway. To the left, ligand concentrations of *[L]=94μM* and *[L]=720μM* were used, which are in the adaptive region $K_I << L << K_A$. To the right ligand concentrations of *[L]= 720μM* and *[L]=5760μM*, outside the adaptive region. Notice the large overlap of the distributions. This effect reduces the memory capacity of *E. Coli*.

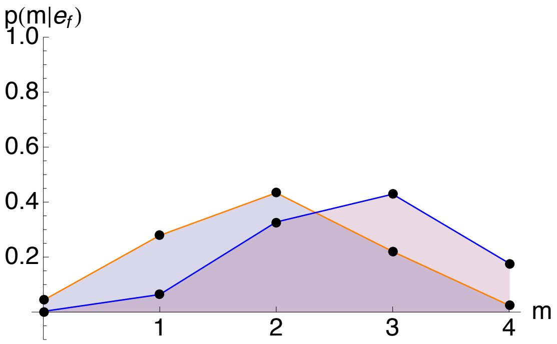

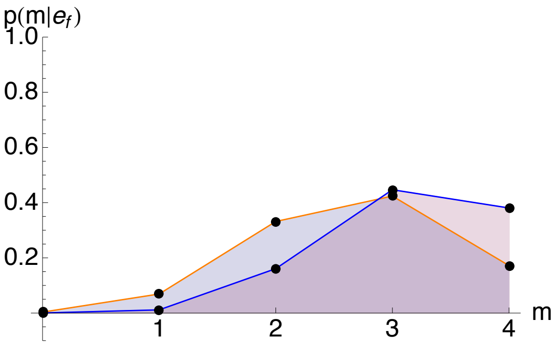

Supplement: S3 Figure — Probability distributions of the methylation level for low and high signals. Probability distribution of methylation levels for low (orange) and high (blue) ligand concentration levels in the chemotaxis pathway. To the left, ligand concentrations of [L] = 94µM and [L] = 720µM were used, which are in the adaptive region KI<<L<<KA. To the right ligand concentrations of [L] = 720µM and [L] = 5760µM, outside the adaptive region. Notice the large overlap of the distributions. This effect reduces the memory capacity of E. coli. (PDF) [file pcbi.1003974.s003.pdf]
